# Navigating the depths: an endoscopic triumph in removing a massive duodenal polyp

**DOI:** 10.1055/a-2282-2929

**Published:** 2024-04-03

**Authors:** Fatih Aslan, Orhun Cig Taskin, Serhat Ozer, Bahadir Hakan Oguz

**Affiliations:** 1 587267 Gastroenterology and Advanced Endoscopy, Koc University Hospital, Istanbul, Turkey; 2 587267 Pathology, Koc University Hospital, Istanbul, Turkey; 3 587267 Anesthesiology and Reanimation, Koc University Hospital, Istanbul, Turkey

Endoscopic submucosal dissection (ESD) is a minimally invasive method for treatment of early gastrointestinal (GI) tumors at any site; however, duodenal ESD is technically challenging because of the anatomic features and high risk of complications, including bleeding and perforation 1 . Here, we report a case of en bloc removal of a distal duodenal polyp of 10 cm in length using ESD, followed by endoscopic closure.

A 57-year-old man presented with dyspepsia and fecal occult blood positivity. On upper GI endoscopy, a flat lesion of around 10 cm, with an irregular surface pattern, was noted in the third part of the duodenum, 8 cm distal to the ampulla of Vater ( Fig. 1 ). Mucosal biopsies revealed high grade dysplasia. Magnetic resonance imaging and endoscopic ultrasound were normal, except for duodenal wall thickness. Our local multidisciplinary committee recommended a surgical approach, either a Whipple operation or duodenectomy; however, the patient refused surgery and ESD was used instead ( Video 1 ).

**Fig. 1:**
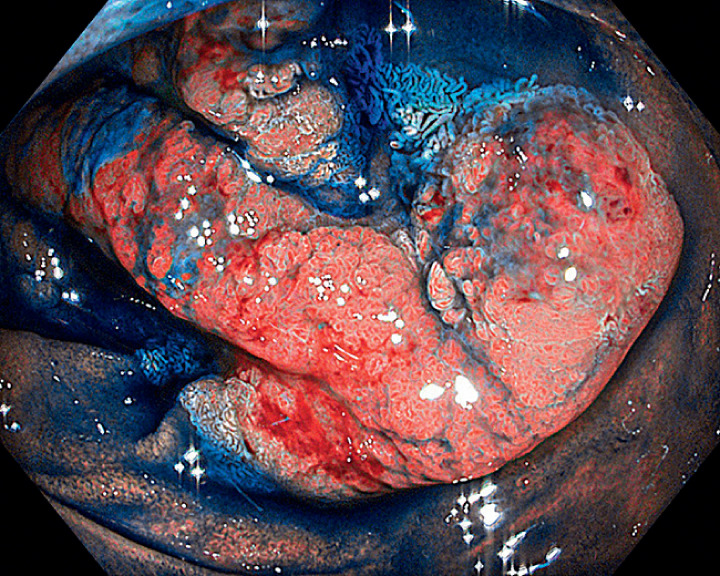
Endoscopic view of the duodenal adenoma after the application of indigo carmine dye.

Endoscopic removal of a giant distal duodenal adenoma.Video 1

ESD was performed using a standard gastroscope with the patient under general anesthesia. The water pressure 2 , single-tunnel 3 , and single-clip traction 4 methods were used ( Fig. 2 ), resulting in en bloc removal of the lesion in 183 minutes. Given the risk of delayed perforation and bleeding, the resection area was closed with a single endoscopic Overstitch suture system, using a double-channel gastroscope ( Fig. 3 ). A nasoenteral tube was placed distal to the resection area and the patient was commenced on an oral diet after 4 hours. He was discharged on the third postoperative day, without experiencing any adverse events. The final pathology report was consistent with a tubulovillous adenoma with high grade dysplastic foci; no invasive cancer was noted ( Fig. 4 ). During follow-up endoscopy after 6 months, there was no evidence of recurrence ( Video 1 ).

**Fig. 2:**
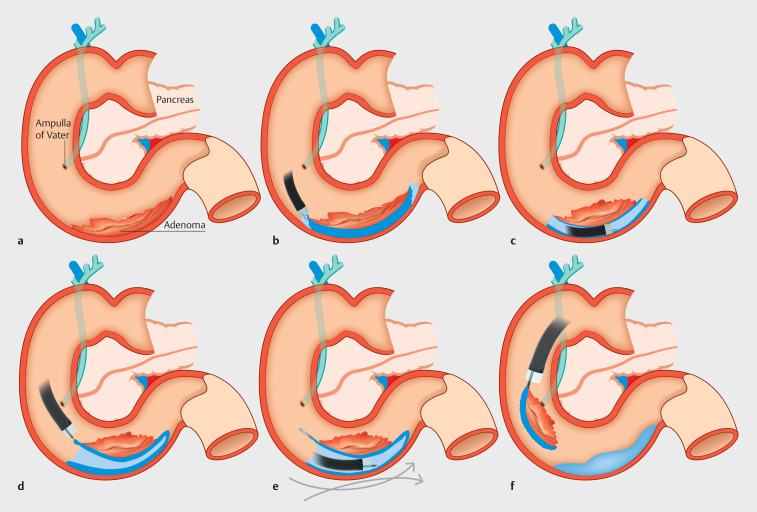
Schematic view of the endoscopic submucosal dissection procedure showing: **a** the adenoma situated in the duodenum; **b** mucosal incision of the distal and proximal parts of the adenoma using the water pressure technique; **c** submucosal dissection of the adenoma using the single-tunnel technique; **d** left and right lateral mucosal incisions; **e** traction applied to the adenoma using the clip-traction technique; **f** endoscopic removal of the adenoma.

**Fig. 3:**
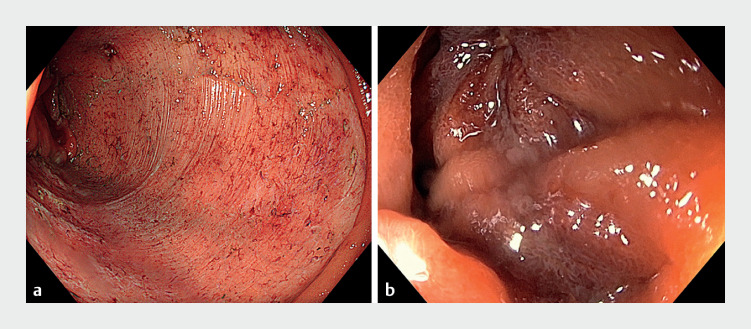
Endoscopic views of: **a** the resection area after completion of the endoscopic submucosal dissection; **b** the resection area following closure with the Overstitch suturing system.

**Fig. 4:**
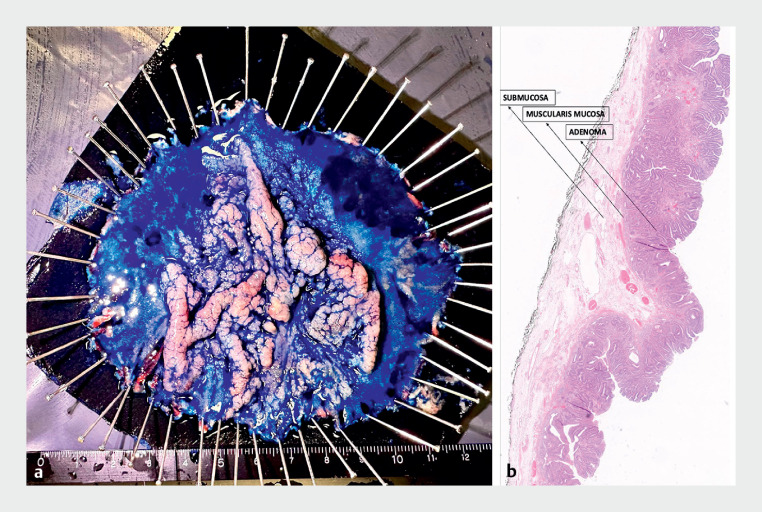
Pathologic examination of the excised adenoma showing: **a** the macroscopic appearance; **b** the histologic appearance on hematoxylin and eosin (H&E) staining, which was consistent with an adenoma (magnification, × 2).

In conclusion, distal duodenal ESD, when combined with certain methods, is a safe and effective method in experienced hands and can be a reasonable alternative to surgery. In addition, we are of the opinion that the endoscopic Overstitch system can prevent delayed complications, even for large resection areas, and that early enteral feeding helps faster recovery.

Endoscopy_UCTN_Code_TTT_1AO_2AG_3AD

## Notes

Endoscopy E-Videos https://eref.thieme.de/e-videos
*E-Videos* is an open access online section of the journal *Endoscopy* , reporting on interesting cases and new techniques in gastroenterological endoscopy. All papers include a high-quality video and are published with a Creative Commons CC-BY license. Endoscopy E-Videos qualify for HINARI discounts and waivers and eligibility is automatically checked during the submission process. We grant 100% waivers to articles whose corresponding authors are based in Group A countries and 50% waivers to those who are based in Group B countries as classified by Research4Life (see: https://www.research4life.org/access/eligibility/ ). This section has its own submission website at https://mc.manuscriptcentral.com/e-videos .
